# Algorithmic management is associated with psychological distress, musculoskeletal pain, and occupational accidents: a cross-sectional study in logistics

**DOI:** 10.1007/s00420-025-02180-5

**Published:** 2025-11-18

**Authors:** Karin Hennum Nilsson, Theo Bodin, Pille Strauss, Nuria Matilla-Santander, Kathryn Badarin, Emma Brulin, Carin Håkansta

**Affiliations:** 1 Unit of Occupational Medicine, Institute of Environmental Medicine, Karolinska Institutet, Torsplan, Solnavägen 4, 113 65 Stockholm, Sweden; 2 Centre for Occupational and Environmental Medicine, Stockholm Region, Torsplan, Solnavägen 4, 113 65 Stockholm, Sweden; 3 Barcelona Biomedical Research Park (PRBB), Barcelona Institute for Global Health (ISGlobal), Doctor Aiguader, 88, 08003 Barcelona, Spain; 4 Universitat Pompeu Fabra (UPF), Edifici de La Mercè, Carrer de La Mercè, 12, 08002 Barcelona, Spain; 5 Faculty of Health and Life Sciences, School of Sport, Nutrition and Allied Health Professions (SNAHP), Oxford Brookes University, Oxford, UK; 6 Department of Working Life Science, Karlstad Business School, Karlstad University, 651 88 Karlstad, Sweden

**Keywords:** Algorithmic management, Occupational safety and health, Logistics, Digitalization, Psychological distress, Occupational accidents, Workplace injuries, Musculoskeletal pain

## Abstract

**Objective:**

Algorithmic Management (AM) is increasingly shaping work environments across various sectors, influencing how tasks are assigned and monitored. While concerns have been raised regarding its potential impact on worker health, empirical evidence remains limited. This study examines the association between level of AM exposure and adverse health outcomes among logistics workers.

**Methods:**

This cross-sectional study used an online survey, targeting logistics workers in Sweden. AM exposure was measured using an 11-item scale capturing aspects such as task allocation, surveillance, and performance monitoring. Health outcomes included psychological distress, musculoskeletal pain, headaches, sleep disturbances, and occupational accidents.

**Results:**

Higher AM exposure was associated with increased prevalence of psychological distress (PR 2·12, 95% CI 1·49–3·02), occupational accidents (PR 1·92, 95% CI 1·22–3·01), headaches (PR 1·68, 95%CI 1·09–2·58), and musculoskeletal pain (PR 1·54, 95% CI 1·23–1·92). Stratified analyses revealed stronger associations for drivers, particularly regarding psychological distress, headaches, and sleep disturbances, while warehouse workers exhibited less consistent patterns.

**Conclusions:**

These findings highlight AM as a potential occupational health hazard, particularly when involving high levels of automated oversight and direction. While AM can enhance efficiency, its impact on worker well-being and public health warrants further attention and potentially mitigation strategies to inform policies that balance technological advancements with worker health protection.

## Background

The organization of work through digital technologies has significant implications for public health (Frank et al. 2023) and Algorithmic Management (AM) is increasingly prevalent across various sectors in both platform and non-platform work (Milanez et al. 2025; Wood 2021). AM refers to digital technologies driven by algorithms that assume managerial functions (Lee et al. 2015) and is used for purposes such as directing and evaluating workers or providing data-driven insights and recommendations, thus automating or augmenting functions previously carried out by managers (Baiocco et al. 2022; Fernández-Macías et al. 2023; Kellogg et al. 2019; Mateescu & Nguyen 2019; Urzi Brancati et al. 2020; Wood 2021). Direction involves task allocation and instructions on task completion through automated decision-making. Evaluation relies on continuous data collection on productivity, with assessments provided as feedback to workers and managers (Fernández-Macías et al. 2023). According to a report from the Organization for Economic Co-operation and Development (OECD), in the United States, 90% of firms have adopted at least one algorithmically driven tool for instructing, monitoring, or evaluating workers. Similarly, across the European countries included in the survey, the average adoption rate is 79% (Milanez et al. 2025). The extent of workers’ exposure to AM practices and the specific managerial functions controlled by algorithms vary widely across companies, industries, and national contexts (Milanez et al. 2025). The logistics sector exemplifies a comprehensive implementation of AM, where algorithms commonly manage task assignments and monitor worker performance to a high degree (Wood 2021).

Workplace conditions shaped by managerial practices, including the adoption of technological innovations, have been recognized as critical elements in shaping health disparities (Peters et al. 2022). It has been argued that AM resembles earlier regimes of strict managerial control with negative implications for worker’s well-being, referring to AM as “Digital Taylorism” (Howard 2022; Liu 2023; Noponen et al. 2023). Kellogg et al. (2019) highlight the intrusive nature of AM, arguing that it presents a more comprehensive, opaque, instantaneous, and interactive form of technical control than previous regimes, and stressing the implications this could have on workers’ health. Research focusing on perceptions and job quality of workers managed by algorithms claims that AM could influence various dimensions of job quality that are linked to health, such as workload, task significance, job security, social support and interpersonal relations, decision authority, organizational trust, and opportunities for development (Baiocco et al. 2022; Christenko et al. 2022; Fernández-Macías et al. 2023; Howard 2022; Parent-Rocheleau and Parker 2022; Urzí Brancati and Curtarelli 2021; Vignola et al. 2023). Additionally, the innate task standardization and efficiency maximization of AM can exacerbate existing health inequities by disproportionately affecting workers in precarious positions (Peters et al. 2022). The introduction of AM in a workplace may thus also introduce rising demands on workers, coupled with a decrease in available resources (Parent-Rocheleau and Parker 2022). This aligns with the Job Demands–Resources (JD-R) model developed by Demerouti et al. (2001), who suggest that occupational health—and burnout in particular—result from an imbalance between job demands and the resources available to workers. The JD-R framework thus provides a strong rationale for examining not only the job quality for workers under AM, but also the health outcomes of exposure to AM.

Public health literature increasingly recognizes the role of workplaces and managerial practices as critical determinants of health (Rugulies et al. 2023), with a need for a systemic integrated approach at both policy and enterprise level to address organizational conditions influencing health disparities (Kaplan 2006; Peters et al. 2022; Rugulies et al. 2023). While concerns have been raised, empirical evidence linking AM to work-related health outcomes remains scarce. The limited evidence indicates that when AM is used to monitor performance and dictate work content, it can have negative effects on workers’ health (Hill 2021; Kinowska and Sienkiewicz 2023; Peng et al. 2024; Semujanga and Parent-Rocheleau 2024). However, since the cited studies primarily focus on platform workers (Hill 2021; Peng et al. 2024; Semujanga and Parent-Rocheleau 2024) or rely on safety representatives and managers as proxies for affected workers rather than direct accounts (Kinowska and Sienkiewicz 2023), there remains a critical need for research examining the impact of AM on the occupational health of standard-employed workers.

This study uses the logistics sector as a context to investigate the association between exposure to AM and health among workers, e.g. drivers and warehouse workers. The health outcomes of interest are psychological distress, musculoskeletal pain, headaches, sleep disturbance, and injuries.

## Method

### Data source and study population

This cross-sectional study is based on survey data collected within the AMOSH (Algorithmic Management and Occupational Safety and Health) project that ran 2022–2024. The study was approved by The Swedish Ethical Review Authority (Dnr 2022-00169; Dnr 2023-06401-02).

Recruitment and data collection via an on-line survey (appended as supplementary material) took place between February and July 2024. The study participants were recruited using online marketing campaigns on social media (Facebook and Instagram), targeting workers in logistics and warehousing. No compensation was paid to the participants. Upon request, two trade unions disseminated information about the study to their members via newsletters.

**Fig. 1:**
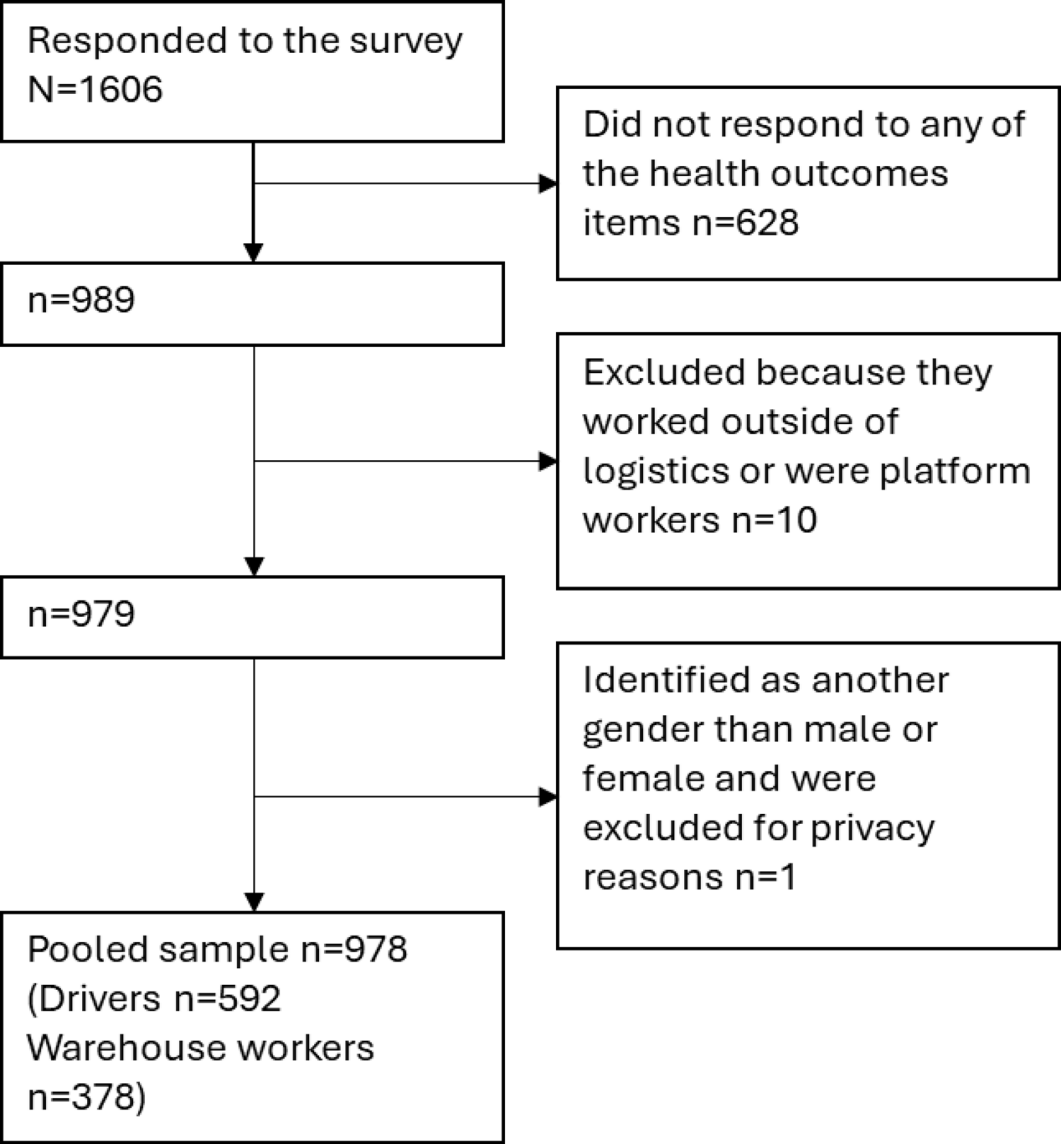
Participant flowchart showing exclusions and the final analytical sample (n = 978)

A total of 1606 responded to the survey. Inclusion criteria included those currently living and working in Sweden and who were actively working in warehousing or as drivers within distribution, and in total 989 respondents completed the survey. Respondents who had missing values on all the health outcomes items were excluded, as were seven platform workers. Furthermore, for the purposes of this analysis, three participants working in sectors other than logistics were excluded. One individual reported to be another gender than male or female and was subsequently not included for privacy reasons. The final analytical sample consisted of 978 employed workers (Fig. 1).

### Variables

#### Exposure: algorithmic management (AM)

AM was assessed using 11 items that captured different aspects of workplace algorithmic control, including automated task allocation and instructions, digital surveillance, automated shift allocation, automated performance monitoring and feedback, and automated job termination. An extra question only applied to drivers: monitoring of driving style. The instrument was created by the research group and incorporated items from the questionnaire used in the Algorithmic Management and Platform Work (AMPwork) survey (Fernández-Macías et al. 2023) and the Algorithmic Management Questionnaire (AMQ) (Parent-Rocheleau et al. 2024). The wording of some questions was adjusted to fit our population in logistics in Sweden. A factor analysis was performed on the AM scale, which indicated a unified construct (Factor 1, Eigenvalue 2·62 Factor 2, Eigenvalue 0·33). Tests to assess the internal consistency of the scale showed good reliability with a Cronbach’s alpha of 0·81.

An AM index was created by summing the values across the AM exposure variables to provide an overall measure of AM, with higher scores indicating greater levels of AM. For warehouse workers, the scores ranged from 0 to 30, and for drivers, 0–33. The score sum was divided into equal groups based on total scores: 0–10, 11–20 and 21–30 for warehouse workers and 0–11, 12–22, 23–33 for drivers. These groups were categorized “No/Low,” “Moderate,” and “High” levels of AM. The group No/Low was used as the reference group for all the analyses.

#### Outcome variables: health

Five health outcomes were investigated. Psychological distress was assessed using the Kessler-6 (K6) scale (Kessler et al. 2002), which measures symptoms of depression, nervousness, hopelessness, restlessness, worthlessness, and effort-related distress. Responses were on a 5-point Likert scale (0–4). A total score (sum of the six items) of above 13 indicated severe psychological distress.

Workplace injuries were defined as incidents occurring in connection with work that resulted in personal injury or illness and required at least one day of sick leave in the past 12 months. The question was adapted and modified from the Swedish national Work-related Disorders Survey (Arbetsmiljöverket 2023).

Musculoskeletal pain was categorized based on frequency of pain reported in the shoulders or neck, arms, or back with three items. Reporting pain occurring more than once a week was categorized as frequent musculoskeletal pain. This measure is adapted from the Stockholm Public Health Cohort Study, a well-established survey designed to track health trends and risk factors in the population (Svensson et al. 2013). Headaches and eyestrain were assessed similarly to musculoskeletal pain.

Sleep disturbances were assessed using a single item from the COPSOQ III (Copenhagen Psychosocial Questionnaire) (Burr et al. 2019), a validated tool for assessing psychosocial work environments and their impact on health outcomes. Sleep disturbances were classified as present if reported most or all of the time in the past four weeks.

#### Covariates

Covariates were selected a priori, based on theoretical considerations and the limited existing literature. They were included if they were plausibly associated with both the exposure and the outcomes without being on the causal pathway. No statistical selection methods were used to determine which variables to include in the models. We used the same set of covariates across all models. Age was grouped into four groups, including age 18–35, 36–45, 46–55 and above 55. Gender was recorded with the options Woman, Man, or Other. Country of birth was categorized into OECD countries and non-OECD countries.

Income levels were divided into four categories; 25,000 SEK or less, 25,500–35000 SEK, 35,500–45000 SEK or 45,500 or above (1 EUR ≈ 11.5 SEK, 1 USD ≈ 10.5 SEK, mid-2024 rates). Educational attainment was classified into three categories: Less than compulsory school, High school diploma, Bachelor’s degree or higher.

Workplace tenure was recorded in five categories: less than one year, 1–3 years, 4–6 years, 7–10 years, and more than 10 years. Respondents also indicated whether they were members of a trade union. Company size was categorized based on the number of employees, with groups ranging from 1 to 10, 11 to 100, and more than 100 employees. Employment type was classified into Permanent contract, Fixed-term, or Trial period. Additionally, contracted working hours were reported as Full-time, Part-time (minimum 75 percent) or Part-time (50 percent or less).

The whole questionnaire was tested through cognitive interviews on eight drivers and warehouse workers to make sure the questions were valid and easy to understand. The cognitive interviews did not result in any questions being removed, but wording was revised in some instances.

### Statistical analysis

To address missing data, we applied multiple imputation using a fully conditional specification (chained equations) approach. The imputation model included all variables used in the analysis to ensure consistency between imputation and analysis models. Missing data were generally few, with dependent variables having 1 percent missing values. Most independent variables had minimal missing data (≤ 5 percent), while age unexpectedly had 19 percent missing, possibly due to a user interface glitch in the online survey.

We used predictive mean matching with the five nearest neighbors for imputation and generated 30 imputed datasets. All analyses, including Poisson regression models, were conducted in Stata, version 16, with a random seed set to ensure reproducibility of the imputation process.

The prevalence of health outcomes, socio-demographic, and employment characteristics was calculated by levels of exposure to AM. We conducted a pooled analysis including both drivers and warehouse workers. Given the known contextual and exposure differences between these groups, we also performed stratified analyses by worker type to explore whether the observed associations were consistent across groups.

The associations between AM and the five health outcomes were examined using Poisson regression models using robust standard errors. Prevalence Ratios (PRs) were estimated with 95 percent Confidence Intervals (CI’s). The AM index was the main independent variable of interest, inserted as a categorical variable of No/low, Moderate and High exposure to AM. Adjusted models included covariates for age, gender, country of birth, education level, income, company size, tenure, employment type, contracted hours, and union membership.

To test the robustness of our findings, we conducted a sensitivity analysis using the AM index as a continuous variable (range 0–33) to examine dose–response relationships.

Potential multicollinearity among covariates in the adjusted models was assessed by calculating pairwise Pearson correlation coefficients and variance inflation factors (VIFs). Where high collinearity was detected (VIF values > 5), we conducted sensitivity analyses by removing the variable from the fully adjusted model.

## Results

### Sample characteristics

The sociodemographic and employment characteristics of the sample are presented in Table 1 and Table 2, stratified by exposure to AM. A total of 978 participants completed the survey. The sample consisted predominantly of men (78 percent), and 89 percent had at least a high school diploma. Most respondents were employed on permanent contracts (91 percent), and 74 percent reported being union members. The majority (66 percent) reported a monthly income of 25,500–35,000 SEK.

**Table 1:** Sociodemographic characteristics of the sample, stratified by level of exposure to AM, in total (*n* = 978) and in %

	Exposure to algorithmic management			
	No/low n (%)	Moderate n (%)	High n (%)	Total n (%)
Exposure to algorithmic management	553 (57.0)	333 (34.3)	84 (8.7)	978
Age				
18–35 years	154 (53.3)	114 (39.4)	21 (7.3)	289 (36.9)
36–45 years	108 (53.2)	74 (36.5)	21 (10.3)	203 (25.9)
46–55 years	81 (54.4)	49 (32.9)	19 (12.8)	149 (19.0)
56–68 + years	98 (68.5)	40 (28.0)	5 (3.5)	143 (18.2)
Gender				
Men (78%)	439 (57.8)	252 (33.2)	68 (9.0)	759 (78.2)
Women (22%)	114 (54.0)	81 (38.4)	16 (7.6)	211 (21.8)
Education				
Up to compulsory school	48 (45.7)	37 (35.3)	20 (19.0)	105 (11.0)
High school diploma	465 (58.9)	271 (34.4)	53 (6.7)	789 (82.5)
Bachelor’s degree or higher	32 (51.6)	21 (33.9)	9 (14.5)	62 (6.5)
Country of birth				
OECD	520 (60.2)	287 (33.2)	57 (6.6)	864 (90.0)
Non-OECD	30 (31.3)	41 (42.7)	25 (26.0)	96 (10.0)

Missing values in the table: Age: 193 missing. Education: 24 missing. Country of birth: 18 missing.

**Table 2:** Employment and company characteristics, stratified by level of exposure to AM, in total (*n* = 978) and in %

	Exposure to algorithmic management			
	No/low n (%)	Moderate n (%)	High n (%)	Total n (100%)
Exposure to algorithmic management	553 (57.0)	333 (34.3)	84 (8.7)	978
Employment type				
Permanent contract	506 (58.3)	294 (33.9)	68 (7.8)	868 (91.2)
Fixed-term contract	21 (42.9)	21 (42.9)	7 (14.3)	49 (5.2)
Trial period	14 (41.2)	14 (41.2)	6 (17.7)	34 (3.6)
Contracted hours				
Full-time	501 (57.1)	307 (35.0)	69 (7.9)	877 (92.5)
Part-time, min 75%	17 (51.5)	11 (33.3)	5 (15.2)	33 (3.5)
Part-time, 50% or less	20 (52.6)	11 (28.9)	7 (18.4)	38 (4.0)
Tenure (current employer)				
< 1 year	54 (49.1)	46 (41.8)	10 (9.1)	110 (11.5)
1–3 years	139 (51.9)	99 (36.9)	30 (11.2)	268 (28.1)
4–6 years	118 (62.8)	50 (26.6)	20 (10.6)	188 (19.7)
7–10 years	75 (57.3)	48 (36.6)	8 (6.1)	131 (13.7)
10 years <	156 (60.9)	87 (34.0)	13 (5.1)	256 (26.9)
Size of company				
1–10 employees	49 (52.1)	35 (37.2)	11 (11.7)	94 (9.7)
11–100 employees	336 (66.1)	142 (28.0)	30 (5.9)	508 (52.4)
100 employees <	168 (45.8)	155 (42.2)	44 (12.0)	367 (37.9)
Income (sek/month)				
25,000 or less	35 (53.8)	21 (32.3)	9 (13.8)	65 (6.8)
25,500–35,000	351 (55.7)	223 (35.4)	56 (8.9)	630 (66.2)
35,500–45,000	104 (59.1)	60 (34.1)	12 (6.8)	176 (18.5)
45,000 <	54 (67.5)	21 (26.3)	5 (6.3)	80 (8.4)
Union member				
Yes	403 (58.2)	233 (33.7)	56 (8.1)	692 (74.4)
No	130 (54.6)	85 (35.7)	23 (9.7)	238 (25.6)

Missing values in the table: Type pf employment: 27 missing. Contracted hours: 30 missing. Tenure: 25 missing. Size of company: 9 missing. Income: 27 missing/prefer not to answer. Union membership: 48 missing/prefer not to answer.

In terms of AM exposure, 57 percent of the participants were in the no/low category, 34 percent in the moderate, and 9 percent in the high exposure category. Thus, 43 percent of the workers were moderately or highly exposed to AM, according to our measurements. Moderate and high exposure levels were more common among the younger participants, those born outside of the OECD, and those with lower education. However, no statistical tests were conducted to determine whether these group differences were significant.

In the total sample, 23 percent of the workers reported psychological distress, 16 percent had experienced an occupational accident which required sick leave, 29 percent reported sleep disturbances, 42 percent experienced frequent musculoskeletal pain, and 18 percent reported frequent headaches.

### Associations between algorithmic management and health outcomes

The pooled analysis, which includes both drivers and warehouse workers, shows a stepwise increase in the likelihood of adverse health outcomes for individuals in moderate and high exposure categories. This indicates a potential dose–response relationship. The analysis includes PRs and 95 percent CIs for moderate and high levels of exposure to AM compared to the reference group; those with low or no exposure. The crude and adjusted results of the analysis examining the association between exposure to AM and the five health outcomes are presented in Table 3. Bolded values indicate statistical significance at *p* < 0.05.

**Table 3:** Crude and adjusted prevalence ratios (PRs) with 95% confidence intervals (CI’s) for self-reported health and injuries in association with algorithmic management. Significant values in bold

	Exposure to algorithmic management				
	No/Low	Moderate		High	
		PR	CI 95%	PR	CI 95%
Psychological distress					
CRUDE	1 (ref)	**1**.**61**	**1**.**22–2**.**13**	**2**.**75**	**2**.**05–3**.**70**
ADJUSTED	1 (ref)	**1**.**50**	**1**.**13–1**.**98**	**2**.**12**	**1**.**49–3**.**02**
Occupational accidents					
CRUDE	1 (ref)	**1**.**56**	**1**.**13–2**.**15**	**1**.**98**	**1**.**31–2**.**98**
ADJUSTED	1 (ref)	**1**.**48**	**1**.**07–2**.**05**	**1**.**92**	**1**.**22–3**.**01**
Musculoskeletal pain					
CRUDE	1 (ref)	**1**.**21**	**1**.**03–1**.**44**	**1**.**58**	**1**.**30–1**.**93**
ADJUSTED	1 (ref)	**1**.**21**	**1**.**02–1**.**43**	**1**.**54**	**1**.**23–1**.**92**
Sleep disturbances					
CRUDE	1 (ref)	**1**.**34**	**1**.**07–1**.**68**	**1**.**58**	**1**.**18–2**.**12**
ADJUSTED	1 (ref)	1.25	0.99–1.56	1.30	.92–1.82
Headaches					
CRUDE	1 (ref)	1.26	0.91–1.74	**1**.**91**	**1**.**31–2**.**79**
ADJUSTED	1 (ref)	1.15	0.83–1.59	**1**.**68**	**1**.**09–2**.**58**

Adjusted models control for age, gender, country of birth, education, income, company size, tenure, employment type, contracted hours, and union membership.

#### Psychological distress

As shown in Table 3, the pooled analysis revealed that both moderate and high AM exposure were significantly associated with psychological distress (adjusted PR = 1.50, 95% CI:1.13–1.98 for moderate; 2.12, 95% CI:1.49–3.02 for high).

**Fig. 2:**
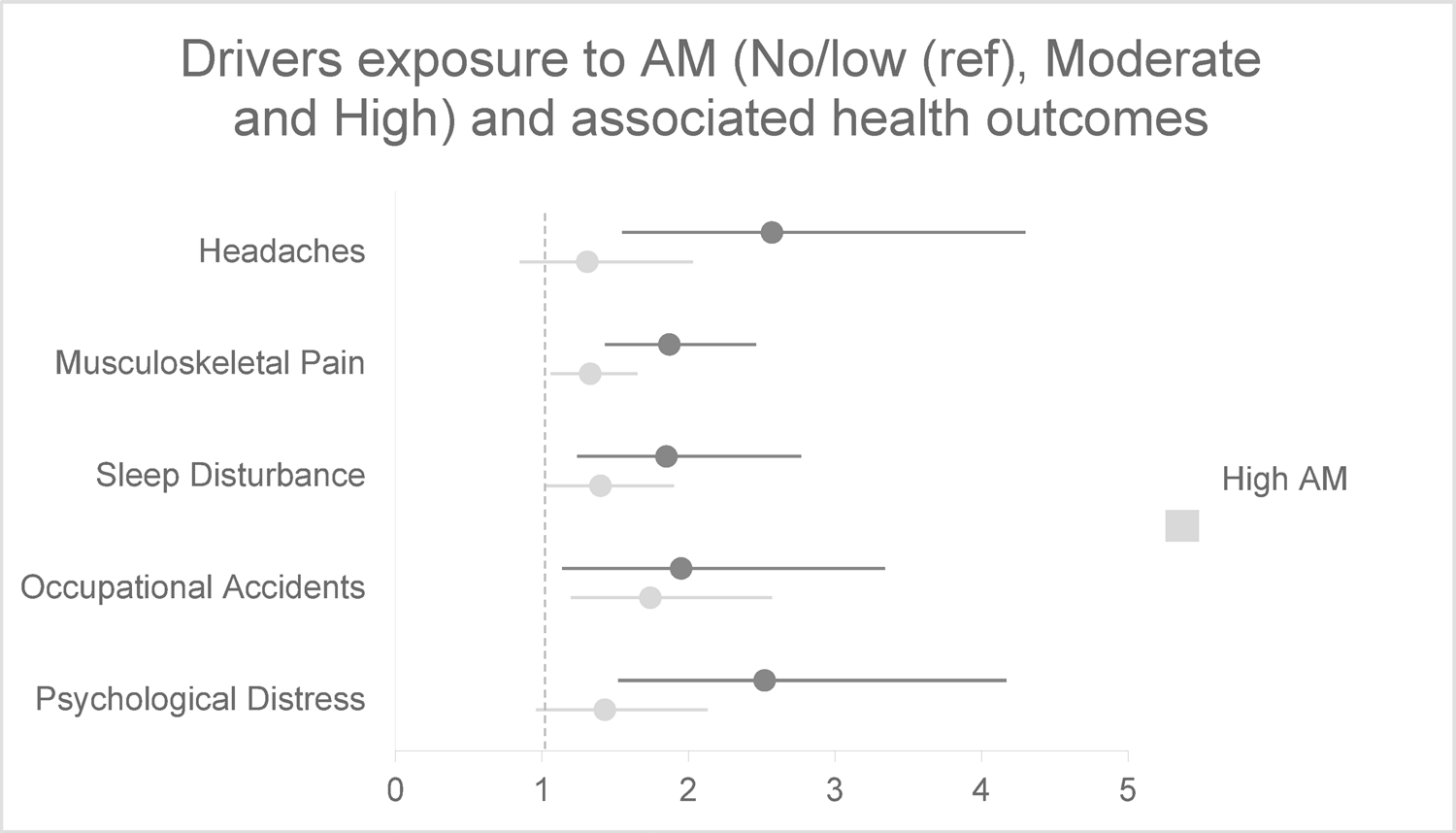
Adjusted results of self-reported health and injuries among drivers (n = 592) in association with algorithmic management. Adjusted models control for age, gender, country of birth, education, income, company size, tenure, employment type, contracted hours, and union membership

**Fig. 3:**
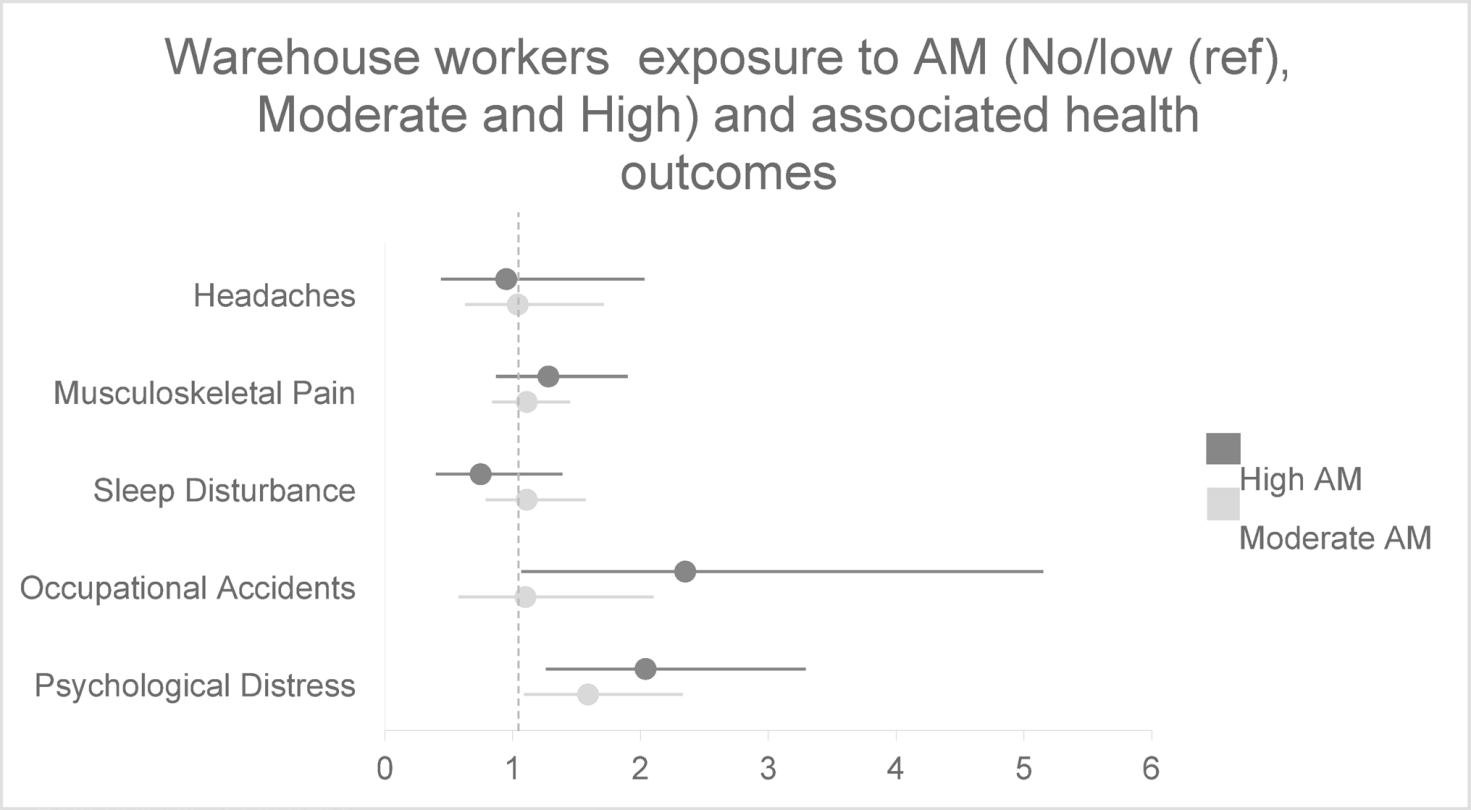
Adjusted results of self-reported health and injuries among warehouse workers (n = 378) in association with algorithmic management. Adjusted models control for age, gender, country of birth, education, income, company size, tenure, employment type, contracted hours, and union membership

This pattern was consistent in the stratified analyses. Among drivers (Fig. 2), both moderate and high AM exposures were significantly associated with psychological distress. Similarly, among warehouse workers (Fig. 3), both exposure levels were associated with a significantly increased risk of psychological distress.

#### Occupational accidents

In the pooled model (Table 3), high AM exposure was associated with a significantly higher prevalence of occupational accidents (adjusted PR = 1.92, 95% CI:1.22–3.01).

Among drivers (Fig. 2), both moderate and high exposure were associated with increased prevalence of accidents. Among warehouse workers (Fig. 3), only high exposure was significantly associated with occupational accidents.

#### Musculoskeletal pain

Musculoskeletal pain was also significantly associated with AM exposure in the pooled analysis (Table 3), particularly at high levels (adjusted PR = 1.54, 95% CI:1.23–1.92).

In the stratified analysis for drivers (Fig. 2), this association remained significant. For warehouse workers (Fig. 3), no statistically significant associations were observed between AM and musculoskeletal pain.

#### Sleep disturbances

Although the crude model showed an association, this was not statistically significant after adjustment in the pooled sample (Table 3) (adjusted PR for high AM exposure = 1.30, 95% CI:0.92–1.82).

Among drivers (Fig. 2), both moderate and high exposure were significantly associated with sleep disturbances. Among warehouse workers (Fig. 3), no significant associations were found.

#### Headaches

In the pooled analysis (Table 3), high AM exposure was associated with frequent headaches (adjusted PR = 1.68, 95% CI:1.09–2.58).

This association was mainly driven by the driver subsample (Fig. 2), where a significant association was observed for high AM exposure. Among warehouse workers (Fig. 3), no significant associations were found between AM exposure and headaches.

#### Sensitivity analyses

A dose–response trend was observed across outcomes in the sensitivity analysis using the continuous AM scale. Higher AM scores were associated with a greater prevalence of psychological distress (PR = 1.038), occupational accidents (PR = 1.034), and musculoskeletal pain (PR = 1.020). Associations with headaches and sleep disturbances did not reach statistical significance.

*Multicollinearity assessment*: Correlations between variables were moderate to low, for example the correlation between age and tenure was r = 0.33, *p* < 0.001; between education and income, r = − 0.02, *p* < 0.05. Most VIFs were < 5, apart from Education and Country of birth. Excluding Education had little impact (biggest increase was for musculoskeletal pain: PR 1.53 to 1.62 for high exposure), while excluding Country of birth produced a somewhat larger change for psychological distress (biggest increase PR 2.12 to 2.38 for high exposure). Overall, however, the results were robust to these adjustments, resulting in modest increases of associations.

## Discussion

The results of this study reveal significant associations between exposure to AM and various health outcomes, with notable differences in the magnitude and patterns of these associations across exposure levels and occupational groups. Overall, higher levels of AM exposure were linked to a greater likelihood of adverse health outcomes, particularly concerning psychological distress, occupational accidents, and musculoskeletal pain, while its association with headaches and sleep quality was inconclusive. These findings suggest that AM practices, especially at high exposure levels, pose a meaningful occupational health risk, particularly through their influence on psychological well-being, injuries, and physical strain. Drivers appear to experience more pronounced health issues, particularly regarding psychological distress, headaches, and sleep disturbances. For warehouse workers, the association between AM exposure and health outcomes was overall weaker and conclusive only for psychological distress and occupational injuries. This may reflect differences in how AM is implemented, which aspects of AM are most prominent in each occupational group, or inherent differences between the two types of work. It should also be noted that when stratifying the sample by occupation, the groups were relatively small (378 warehouse workers and 592 drivers), resulting in less power and, in some cases, wide CI’s.

Our findings align with theoretical perspectives suggesting that AM restructures work in ways that may increase job demands while limiting access to critical job resources. The JD-R model (Demerouti et al. 2001) provides a useful lens for understanding these dynamics. AM practices, such as intensified surveillance, automated task allocation, and performance monitoring, have been theorized to increase psychological and physical demands on workers, which could contribute to adverse health outcomes (Kinowska and Sienkiewicz 2023; Urzí Brancati and Curtarelli 2021; Vignola et al. 2023). Previous empirical research has raised concerns about how AM intensifies workload, standardization, and work pace, leading to increased stress and reduced well-being (Delfanti 2021; Kinowska and Sienkiewicz 2023).

Furthermore, AM may also shape the availability of job resources that can counterbalance the increase in demands. Autonomy, social support, and organizational trust, which are framed as protective factors in the JD-R model, have been described as potentially weakened by AM practices (Jarrahi et al. 2021; Vignola et al. 2023).

Our findings—showing that higher exposure to algorithmic management is associated with increased prevalence of psychological distress, occupational injuries, and musculoskeletal pain—may reflect these heightened demands and reduced resources placed on workers. The demands could compel employees to intensify their work pace while deprioritizing safety measures that protect both their physical and mental well-being. These results align with growing concerns about the risks posed by algorithmic control and may serve as a foundation for future research into the pathways linking algorithmic management to worker health.

As one of the first studies of AM outside the platform economy, our study directly links AM exposure to adverse self-reported health outcomes in a logistics workforce. In our study, almost half of the population had moderate or high exposure to AM, indicating that AM exposure is prevalent within logistics. Although the occupations we investigated were within the same sector, the observed differences between them suggest that certain dimensions of AM may be more pronounced in some occupations than others. These differences could lead to varying exposures to occupational risk factors, such as an increase in pace and loss of autonomy. Furthermore, some applications of AM may be more closely linked to health risks than others, which warrants further investigation. The occupational differences observed also indicate that the way AM is implemented and used may influence its impact on worker health. Overall, our findings highlight the importance of considering the context-specific nature of AM when evaluating its effects on worker health.

### Limitations and future research

As this is a cross-sectional study, we cannot establish causality, and there is a possibility of reverse causality, where workers with pre-existing health challenges may be more likely to work under AM. However, this explanation is less plausible for occupational accidents, which were reported at a higher rate among workers exposed to AM, as these incidents are discrete events rather than ongoing conditions. Additionally, measuring both exposure and outcomes simultaneously and through the same self-reported survey method introduces the risk of common method bias and priming effects, potentially inflating the observed associations. Respondents’ perceptions of AM may be influenced by their current health status, and answering health-related questions immediately after reporting on AM exposure may reinforce a perceived connection between the two. Furthermore, our decision to recruit participants via social media may have introduced selection bias. Workers who experience negative health effects from AM or hold critical views of AM may have been more motivated to participate, potentially skewing the sample toward those with worse health outcomes.

In addition to the many challenges of interpreting cross-sectional studies, especially relying on self-reported exposure and outcomes, this study also measures a relatively new exposure with no common definition or agreed-upon dimensions. Although measures were taken to ensure that the questions were valid and reliable, both through cognitive interviews and factor analysis, there is still a risk that the full experience of being managed by algorithms was not captured and that workers were misclassified regarding their exposure to AM.

Another limitation of this study is the lack of information about the type of establishments where the respondents worked. Although an open-ended question was included asking participants to describe the profile of their workplace, few provided responses. As a result, it is impossible to determine whether respondents with high exposure to AM were, for example, last-mile delivery drivers or worked in the e-commerce sector. The e-commerce sector, known for its intense competition and high-strain working conditions (Gutelius and Pinto 2023; Gutelius and Theodore 2019), often presents a context where workers face greater exposure to strain and where AM may be more deeply embedded in daily operations.

The decision to categorize workers in three levels of exposure to AM may also have affected the analysis. For example, workers who selected the option “Yes, to some extent” to all items (all but one item for the drivers), were categorized as “No/Low” exposure to AM. Similarly, workers who were only exposed to some aspects of AM (such as only surveilled or only directed by algorithms) were categorized as “No/Low”. This may have resulted in a misclassification as, for example, workers experiencing negative effects from being constantly monitored (a central aspect of AM) were categorized as not exposed.

It is also important to acknowledge the presence of multicollinearity among some covariates in the fully adjusted model. This, alongside the other limitations mentioned in our discussion, suggests that the results should be interpreted with some caution.

Future research should investigate the longitudinal health effects of AM to assess causal relationships. Additionally, examining the various functions of AM and their specific impacts on health outcomes would provide deeper insights regarding which aspects of AM are harmful and which should be targeted in future policy recommendations and possible legislation. Additionally, further exploration is needed to map the mediating psychosocial risk factors associated with AM and identify organizational factors that may help mitigate its potentially detrimental effects on worker health.

Our findings suggest a need for organizations to address the occupational safety and health implications of AM systems and for policy makers to introduce policies ensuring that these systems do not exacerbate work-related stress or impair physical health.

## Data Availability

Pseudonymized survey data can be requested from the principal investigator by contacting author CH (carin.hakansta@ki.se), first author KN (karin.nilsson@ki.se), or author TB (theo.bodin@ki.se). Data requests will be pending approval by ethical committees and data sharing agreements.

## Appendix

See Appendix Tables 4, 5, 6, and 7.

**Table 4:** Sociodemographic characteristics of *Drivers*, stratified by level of exposure to AM, in total (*n* = 592) and in %

	Exposure to algorithmic management among drivers			
	No/Low n (%)	Moderate n (%)	High n (%)	Total n (100%)
Exposure to algorithmic management	325 (54.9)	221 (37.3)	46 (7.8)	592
Age				
18–35	93 (54.4)	70 (40.9)	8 (46.8)	171
36–45	53 (48.6)	47 (43.1)	9 (8.3)	109
46–55	44 (46.8)	35 (37.2)	15 (16.0)	94
56–68 +	69 (64.5)	34 (31.8)	4 (3.7)	107
Total	259 (53.8)	186 (38.7)	36 (7.5)	481
Gender				
Men (81%)	269 (55.8)	174 (36.1)	39 (8.1)	482
Women (19%)	56 (50.9)	47 (42.7)	7 (6.4)	110
Total	325 (54.9)	221 (37.3)	46 (7.8)	592
Education				
Up to compulsory school	39 (51.3)	25 (32.9)	12 (15.8)	76
High school diploma	465 (58.9)	271 (34.4)	53 (6.7)	789
Bachelor’s degree or higher	14 (48.3)	10 (34.5)	5 (17.2)	29
Total	322 (54.8)	220 (37.4)	46 (7.8)	588
Country of birth				
OECD	319 (56.1)	210 (36.9)	40 (7.0)	569
Non-OECD	5 (25.0)	9 (45.0)	6 (30.0)	20
Total	324 (55.0)	219 (37.2)	46 (7.8)	589

The table contains missing values: Age: 111 missing. Education: 4 missing. Country of birth: 3 missing.

**Table 5:** Employment and company characteristics for *Drivers*, stratified by level of exposure to AM, in total (*n* = 592) and in %

	Exposure to algorithmic management among drivers					
	No/low n (%)	Moderate n (%)		High n (%)		Total n (100%)
Employment type						
Permanent contract	296 (55.3)		199 (37.2)		40 (7.5)	535
Fixed-term	6 (33.3)		9 (50.0)		3 (16.7)	18
Trial period	13 (54.2)		9 (37.5)		2 (8.3)	34
Total	315 (54.6)		217 (37.6)		45 (7.8)	577
Contracted hours						
Full-time	292 (54.5)		203 (37.9)		41 (7.7)	536
Part-time, min 75%	9 (50.5)		7 (38.9)		2 (11.1)	18
Part-time, 50% or less	11 (52.4)		8 (38.1)		2 (9.5)	21
Total	312 (218)		218 (37.9)		45 (7.8)	575
Tenure (current employer)						
< 1 year	40 (54.1)		27 (36.5)		7 (9.5)	74
1–3 years	76 (46.9)		71 (43.8)		15 (9.3)	162
4–6 years	74 (60.7)		35 (28.7)		13 (10.7)	122
7–10 years	46 (56.8)		32 (39.5)		3 (3.7)	81
10 years <	79 (56.8)		53 (38.1)		7 (5.0)	139
Total	315 (54.5)		218 (37.7)		45 (7.8)	578
Size of company						
1–10 employees	33 (48.5)		30 (44.1)		5 (7.4)	68
11–100	207 (61.1)		109 (32.2)		23 (6.8)	339
100 employees <	85 (46.2)		81 (44.0)		18 (9.8)	184
Total	325 (55.0)		220 (37.2)		46 (7.8)	591
Income (sek/month)						
25,000 or less	11 (45.8)		12 (50.0)		1 (4.2)	24
25,500–35,000	196 (51.3)		153 (40.1)		33 (8.6)	382
35,500–45,000	67 (59.3)		39 (34.5)		7 (6.2)	113
45,000 <	44 (72.1)		13 (21.3)		4 (6.6)	61
Total	318 (54.8)		217 (37.4)		45 (7.8)	580
Union member						
Yes	227 (54.6)		154 (37.0)		35 (8.4)	416
No	82 (55.0)		57 (38.3)		10 (6.7)	149
Total	309 (54.7		211 (37.4)		45 (8.0)	565

The table contains missing values: Employment type: 15 missing. Contracted hours: 17 missing. Tenure: 14 missing. Size of company: 1 missing. Income: 12 missing/prefer not to answer. Union membership: 27 missing/prefer not to answer.

**Table 6:** Sociodemographic characteristics of *Warehouse workers*, stratified by level of exposure to AM, in total (*n* = 378) and in %

	Exposure to algorithmic management among warehouse workers			
	No/low n (%)	Moderate n (%)	High n (%)	Total n (100%)
Exposure to algorithmic management	228 (60.3)	112 (29.6)	38 (10.1)	378
Age				
18–35	61 (51.7)	44 (37.3)	13 (11.0)	118
36–45	55 (58.5)	27 (28.7)	12 (12.8)	94
46–55	37 (67.3)	14 (25.5)	4 (7.3)	55
56–68 +	29 (80.6)	6 (16.7)	1 (2.8)	36
Total	182 (60.1)	91 (30.0)	30 (9.9)	303
Gender				
Men (81%)	170 (61.4)	78 (28.2)	29 (10.5)	277
Women (19%)	58 (57.4)	34 (33.7)	9 (8.9)	101
Total	228 (60.3)	112 (29.6)	38 (10.1)	378
Education				
Up to compulsory school	9 (31.0)	12 (41.4)	8 (27.6)	29
High school diploma	196 (64.1)	86 (28.1)	24 (7.8)	306
Bachelor’s degree or higher	18 (54.6)	11 (33.3)	4 (12.1)	33
Total	223 (60.6)	109 (29.6)	36 (9.8)	368
Country of birth				
OECD	218 (65.1)	93 (27.8)	24 (7.2)	335
Non-OECD	8 (22.2)	16 (44.4)	12 (33.3)	36
Total	226 (60.9)	109 (29.4)	36 (9.7)	371

The table contains missing values: Age: 75 missing. Education: 10 missing. Country of birth: 7 missing.

**Table 7:** Employment and company characteristics of *Warehouse workers*, stratified by level of exposure to AM, in total (*n* = 378) and in %

	Exposure to algorithmic management among warehouse workers					
	No/low n (%)	Moderate n (%)		High n (%)		Total n (100%)
Employment type						
Permanent contract	210 (63.1)		95 (28.5)		28 (8.4)	333
Fixed-term	15 (48.4)		12 (38.7)		4 (12.9)	31
Trial period	1 (10.0)		5 (50.0)		4 (40.0)	10
Total	226 (60.4)		112 (30.0)		36 (9.6)	374
Contracted hours						
Full-time	209 (61.3)		104 (30.5)		28 (8.2)	341
Part-time, min 75%	8 (53.3)		4 (26.7)		3 (20.0)	15
Part-time, 50% or less	9 (52.4)		3 (38.1)		5 (9.5)	17
Total	226 (60.6)		111 (29.8)		36 (9.7)	373
Tenure (current employer)						
< 1 year	14 (38.9)		19 (52.8)		3 (8.3)	36
1–3 years	63 (59.4)		28 (26.4)		15 (14.2)	106
4–6 years	44 (66.7)		15 (22.7)		7 (10.6)	66
7–10 years	29 (58.0)		16 (32.0)		5 (10.0)	50
10 years <	77 (65.8)		34 (29.1)		6 (5.1)	117
Total	227 (60)		112 (29.9)		36 (9.6)	375
Size of company						
1–10 employees	16 (61.5)		5 (19.2)		5 (19.2)	26
11–100	129 (76.3)		33 (19.5)		7 (4.1)	169
100 employees <	83 (45.4)		74 (40.4)		26 (14.2)	183
Total	228 (60.3)		112 (29.6)		38 (10.1)	378
Income (sek/month)						
25,000 or less	24 (58.5)		9 (22.0)		8 (19.5)	41
25,500–35,000	155 (62.5)		70 (28.2)		23 (9.3)	248
35,500–45,000	37 (58.7)		21 (33.3)		5 (7.9)	63
45,000 <	10 (52.6)		8 (42.1)		1 (5.3)	19
Total	226 (60.9)		108 (29.1)		37 (10.0)	371
Union member						
Yes	176 (63.8)		79 (28.6)		21 (7.6)	276
No	48 (53.9)		28 (31.5)		13 (14.6)	89
Total	224 (61.4)		107 (29.3)		34 (9.3)	365

The table contains missing values: Employment type: 4 missing. Contracted hours: 5 missing. Tenure: 3 missing. Income: 7 missing/prefer not to answer. Union membership: 13 missing/prefer not to answer.
